# Circulating trimethylamine‐*N*‐oxide is associated with all‐cause mortality in subjects with nonalcoholic fatty liver disease

**DOI:** 10.1111/liv.14963

**Published:** 2021-06-08

**Authors:** Jose L. Flores‐Guerrero, Adrian Post, Peter R. van Dijk, Margery A. Connelly, Erwin Garcia, Gerjan Navis, Stephan J. L. Bakker, Robin P. F. Dullaart

**Affiliations:** ^1^ Department of Internal Medicine Division of Nephrology University of Groningen University Medical Center Groningen Groningen The Netherlands; ^2^ Department of Internal Medicine Division of Endocrinology University of Groningen University Medical Center Groningen Groningen The Netherlands; ^3^ Laboratory Corporation of America Holdings (Labcorp) Morrisville NC USA

**Keywords:** microbiota, mortality, NAFLD, nonalcoholic fatty liver disease, TMAO, trimethylamine‐*N*‐oxide

## Abstract

**Background and Aims:**

Trimethylamine‐*N*‐oxide (TMAO), a gut microbiota‐liver metabolite, has been associated with cardiometabolic disease. However, whether TMAO is associated with nonalcoholic fatty liver disease (NAFLD) and NAFLD‐related health outcomes remains unclear. We aimed to investigate the association of TMAO with NAFLD and to assess the extent to which the association of TMAO with all‐cause mortality is dependent on the presence of NAFLD in the general population.

**Methods:**

We included 5292 participants enrolled in the Prevention of Renal and Vascular End‐stage Disease (PREVEND) cohort study. Cox proportional‐hazards regression analyses were performed to study the association of TMAO with all‐cause mortality in subjects with and without a fatty liver index (FLI) ≥60, which was used as a proxy of NAFLD.

**Results:**

During a median follow‐up of 8.2 years, 307 subjects died, of whom 133 were classified with NAFLD. TMAO was positively and independently associated with baseline FLI (Std β 0.08, 95% CI 0.05, 0.11, *P* < .001). Higher TMAO was associated with increased risk of all‐cause mortality in subjects with NAFLD, in crude analysis (hazard ratio [HR] per 1 SD, 2.55, 95% CI 1.60, 4.05, *P* < .001) and after full adjustment (_adj_HR 1.90, 95% CI 1.18, 3.04, *P* = .008). Such an association was not present in subjects without NAFLD (crude HR 1.14, 95% CI 0.81, 1.71, *P* = .39; _adj_HR 0.95, 95% CI 0.65, 1.39, *P* = .78).

**Conclusion:**

This prospective study revealed that plasma concentrations of TMAO were associated with all‐cause mortality in subjects with NAFLD, independently of traditional risk factors.

**Abbreviations**
ALT: alanine aminotransferase
AST: aspartate aminotransferase
AU: arbitrary units
BMI: body mass index
CVD: cardiovascular disease
DBP: diastolic blood pressure
eGFR: estimated glomerular filtration rate
FLI: fatty liver index
GGT: gamma‐glutamyltransferase
HDL‐C: high‐density lipoprotein cholesterol
HSI: hepatic steatosis index
HOMA: homeostasis model assessment
HR: hazard ratio
IQR: inter quartile range
NMR: nuclear magnetic resonance
NAFLD: nonalcoholic fatty liver disease
PREVEND: prevention of renal and vascular end‐stage disease
SBP: systolic blood pressure
SD: standard deviation
STROBE: Strengthening the Reporting of Observational Studies in Epidemiology
T2D: type 2 diabetes
TC: total cholesterol
TMAO: trimethylamine‐*N*‐oxide
UAE: urinary albumin excretion
VIF: variance inflation factor

**Key Points**

- Previous studies suggest that TMAO, a gut microbiota‐liver metabolite, may be associated with an increased risk of increased mortality.
- This is the first prospective cohort study to investigate the association between TMAO and risk of all‐cause and cardiovascular mortality in subjects with NAFLD.
- We found that higher concentrations of circulating TMAO is associated with an increased risk of all‐cause mortality in subjects with NAFLD, independently of traditional risk factors and comorbidities.

​

​

## 1 INTRODUCTION

According to the latest reports, nonalcoholic fatty liver disease (NAFLD) has a worldwide prevalence of 25%, being even more prevalent in countries with concomitant obesity, that is, in the United States, where NAFLD affects 30% of the population.^1^ Although the deleterious effect of lipid accumulation in the liver was proposed back in 1849,^2^ the impact of NAFLD as a global health issue and its association with increased risk of mortality was only recognized 150 years later.^3^ The understanding of the aetiology and risk factors of this global health challenge has evolved over the last few years. Recent evidence from in vivo models of NAFLD had pointed to the role of the gut microbiome in the development and progression of NAFLD.^4^, ^5^

It has been proposed that because the liver is the “first pass” organ of gut microbiota‐derived metabolites, it is exposed to the highest concentrations of such metabolites and therefore more vulnerable to their deleterious effects. Similarly, it may be likely that hepatic tissues already affected by inadvertent lipid accumulation are more susceptible to such effects, possibly worsening the clinical prognosis of patients with NAFLD.^6^

Trimethylamine‐*N*‐oxide (TMAO) is a microbiota‐derived metabolite^7^, ^8^ that has recently gained attention as a consequence of its potential role in the development and progression of type 2 diabetes (T2D),^9^ cardiovascular disease (CVD),^10^ and its association with increased mortality risk in the general population.^11^, ^12^ Trimethylamine (TMA) is a by‐product of a microbial fermentation of dietary components such as choline, phosphatidylcholine, and l‐carnitine. Subsequently, TMA is oxidized to TMAO by the liver enzyme flavin monooxygenase 3, whereas circulating TMAO is cleared by the kidneys.^7^

Clinical studies have revealed an association between higher circulating TMAO and NAFLD as well as with nonalcoholic steatohepatitis (NASH).^13^, ^14^, ^15^ Besides the deleterious effects of TMAO in the development of CVD in the context of metabolic disease, TMAO may underlie different mechanisms that affect the clinical course of NAFLD. For instance, it has been reported that TMAO inhibits cholesterol conversion into bile acids, promoting steatosis and worsening the progression of NAFLD.^15^

Given the fact that most patients diagnosed with NAFLD remain asymptomatic, and the progression of the disease is extremely variable,^16^ it is relevant to further investigate whether novel risk factors, that is, microbiota‐derived biomarkers, are associated with and increased risk of mortality in subjects with NAFLD. Therefore, the aim of this study was to interrogate the association of circulating concentrations of TMAO with NAFLD and to assess the extent to which the association of TMAO with all‐cause mortality is dependent on the presence of NAFLD in the general population. Furthermore, considering that the most common cause of death in patients with NAFLD is CVD, patients being twice as likely to die of CVD than of liver disease,^17^ we further explored the association of plasma concentrations of TMAO with cardiovascular mortality in participants with NAFLD.

## 2 METHODS

### 2.1 Study cohort

The Prevention of Renal and Vascular End‐stage Disease (PREVEND) study is a prospective population‐based cohort study in the city of Groningen, The Netherlands. The design of the PREVEND study has been described in detail elsewhere.^18^ Briefly, from 1997 to 1998, all residents from Groningen, excluding pregnant women and people with type 1 diabetes or T2D using insulin, aged 28‐75 years were invited to participate. A total of 40 856 subjects (47.8%) responded to the invitation to participate. From this group, 30 890 subjects had a urinary albumin concentration of <10 mg/L and 9966 subjects had a urinary albumin concentration of ≥10 mg/L in their morning urine sample. After exclusion of subjects with type 1 diabetes and pregnant women, all subjects with a urinary albumin concentration of ≥10 mg/L (n = 7768) and a randomly selected control group with a urinary albumin concentration of <10 mg/L (n = 3395) were invited for further investigations in an outpatient clinic. A total of 8592 individuals completed an extensive examination.

We used data of participants who completed the second screening and were free from liver disease assessed by questionnaire (n = 6894), excluding those with missing samples for assessment of TMAO concentrations (n = 1425) or missing values for assessment of FLI (n = 177), leaving a cohort of 5292 participants with complete information for the analysis. Cases of participants lost to follow‐up were considered as censored cases. This report follows the Strengthening the Reporting of Observational Studies in Epidemiology (STROBE) reporting guideline (Table S1). The protocol for the PREVEND study was approved by the local ethics committee of the University Medical Center Groningen (approval number: MEC96/01/022). All participants provided written informed consent, and all procedures were conducted according to the Declaration of Helsinki.^19^

### 2.2 Laboratory measurements

Laboratory measurements were performed at the Central Laboratory of the University Medical Center Groningen, The Netherlands. Venous blood samples were drawn after an overnight fast while participants rested for 15 min. Ethylenediaminetetraacetic acid (EDTA)‐anticoagulated plasma samples and sera were stored at −80°C until analysis.

TMAO concentrations were measured in EDTA‐anticoagulated plasma samples using a Vantera^®^ Clinical Analyzer (Labcorp), a fully automated, high‐throughput, 400 MHz proton (^1^H) nuclear magnetic resonance (NMR) spectroscopy platform. TMAO was quantified from one‐dimensional (1D) proton (^1^H) Carr–Purcell–Meiboom–Gill (CPMG) spectra by spectral deconvolution algorithm as previously described.^20^, ^21^ The TMAO assay has intra‐assay and interassay coefficients of variation (CV%) range from 4.3% to 10.3% and 9.8% to 14.5%, respectively.^21^

Total cholesterol (TC), triglycerides, and serum creatinine were measured using standard protocols, as described previously.^22^ Serum alanine aminotransferase (ALT) and aspartate aminotransferase (AST) were measured using the standardized kinetic method with pyridoxal phosphate activation (Roche Modular P, Roche Diagnostics). Serum gamma‐glutamyl transferase (GGT) was assayed by an enzymatic colorimetric method (Roche Modular P, Roche Diagnostics). Standardization of ALT, AST, and GGT was performed according to the International Federation of Clinical Chemistry guidelines.^23^, ^24^, ^25^ Urinary albumin excretion (UAE) was measured by nephelometry (Dade Behring Diagnostic) as described in two 24‐hour urine collections, and the results were averaged for analysis. Serum creatinine was measured by an enzymatic method on a Roche Modular analyser (Roche Diagnostics). Serum cystatin C was measured using Gentian Cystatin C Immunoassay (Gentian AS) reagents on a modular analyser (Roche Diagnostics). The estimated glomerular filtration rate (eGFR) was calculated using the Chronic Kidney Disease Epidemiology Collaboration (CKD‐EPI) combined creatinine‐cystatin C Equation.^26^

### 2.3 Clinical measurements

During two outpatient visits, baseline data were collected on demographics, lifestyle factors, anthropometric measurements, medical history, parental history of T2D, and medication use. Information on medication use was combined with information from a pharmacy‐dispensing registry, which had complete information on the drug usage of >95% of subjects in the PREVEND study. Height and weight were measured in standing position without shoes and heavy outer garments. Body mass index (BMI) was calculated as weight (kg) divided by height (metre) squared. Waist circumference was measured as the smallest girth between the rib cage and iliac crest. Systolic and diastolic blood pressure values were measured with an automatic Dinamap XL Model 9300 series device and recorded as the means of the last two recordings of the second visit.

The fatty liver index (FLI) was used as proxy for the diagnosis of NAFLD.^27^, ^28^ The FLI was calculated from BMI, GGT, triglycerides, and waist circumference data according to the following formula: [e (0.953 × loge (triglycerides + 0.139 × BMI + 0.718 × loge (GGT) + 0.053 × waist circumference −15.745)]/[1 + e (0.953 × loge (triglycerides) + 0.139 × BMI + 0.718 × loge (GGT) + 0.053 × waist circumference −15.745)] × 100. The optimum cut‐off value for the FLI is accepted to be 60 with an accuracy of 84%, a sensitivity of 61% and a specificity of 86% for detecting NAFLD as determined by ultrasonography.^27^ FLI ≥60 was therefore used for this study. The FLI is currently considered as one of the best‐validated steatosis scores for larger scale screening studies.^29^ In alternative analyses, we used the hepatic steatosis index (HSI). The HSI is defined as follows: HIS = 8 × ALT/AST ratio + BMI (+2, if diabetes; +2, if female). A cut‐off of HIS ≥ 36 was used as a second proxy of NAFLD).^30^

### 2.4 Ascertainment of end point

Participants were followed from the date of the baseline visit until end of follow‐up (January first 2011). Data on mortality were obtained from the municipal register, and the cause of death was obtained by linking the number of the death certificate to the primary cause of death as coded by a physician from the Central Bureau of Statistics.

### 2.5 Statistical analysis

Data are presented as the mean (SD) or median (interquartile range, IQR) for continuous variables and percentages for categorical variables. Cross‐sectional group differences among FLI groups at baseline were assessed by unpaired *t* tests for normally distributed and loge transformed variables, by Mann–Whitney *U* tests for nonnormally distributed variables or by chi‐squared tests for categorical variables where appropriate. Multivariable linear regression analyses were carried out to disclose the associations of TMAO concentrations with clinical covariates and laboratory parameters, after adjustment for age and sex. To further evaluate whether TMAO was associated with FLI and HSI, two models were built including those variables associated with TMAO and mutually excluding FLI and HSI as well as its determinants. In order to identify the risk of multicollinearity in the multivariable regression analyses, the variance inflation factors (VIFs) were calculated. A high risk of multicollinearity was considered present if the calculated VIF was >5.^31^

For the prospective analysis, the data of the two groups of participants: with NAFLD (n = 1598) and without NAFLD (n = 3694) were analysed separately, given the significant interaction between TMAO and NAFLD (*P*_int_ < .05). We plotted cumulative Kaplan–Meier curves for risk of all‐cause mortality during follow‐up according to tertiles of TMAO, in two groups of participants (with and without NAFLD). Time‐to‐event Cox proportional hazards models were used to compute hazard ratios (HRs) and 95% CI of all‐cause mortality risk in two groups of participants: with NAFLD (n = 1598) and without NAFLD (n = 3694). HRs were calculated in models adjusted for age, sex, T2D medication, smoking behaviour, alcohol consumption, history of cancer, systolic blood pressure, antidiabetic medication, lipid lowering medication, glucose, total cholesterol, high‐density lipoprotein cholesterol (HDL‐cholesterol), albuminuria, and eGFR at baseline. The Cox proportional hazard assumption was tested through the evaluation of independence between scaled Schoenfeld residuals with time for each variable and for every model as a whole; this assumption was met, with no indication for a violation.^32^ In the two groups of participants (with and without NAFLD) the interactions of TMAO with age and eGFR were also evaluated. To further evaluate the robustness of the association and the risk of bias, a sensitivity analysis was conducted to calculate the Robustness of Inference to Replacement.^33^

All statistical analyses were performed with R language for statistical computing software, v. 4.0.3 (2020).^34^

## 3 RESULTS

### 3.1 Baseline characteristics

Out of the 5292 participants with available measurements of TMAO that were included in the current study, 1671 (31.6%) participants had an FLI ≥ 60. Table 1 shows clinical characteristics and laboratory data of the study population according to FLI categorization. Participants with an FLI ≥ 60 were older, more likely to be men and use antihypertensive‐ and lipid‐lowering drugs. Alcohol consumption and cigarette smoking were also more common among participants with elevated FLI. BMI, waist circumference, systolic and diastolic blood pressure, blood glucose, transaminases, GGT, TC, triglycerides, and UAE were higher in participants with an FLI ≥ 60, whereas eGFR and HDL‐cholesterol were lower in participants with an elevated FLI. The median (IQR) plasma total TMAO concentration was 3.58 (2.02, 6.25) μmol/L and 2.99 (1.60, 5.46) in participants with FLI ≥ 60 and <60, respectively (*P* < .001) (Table 1).

**TABLE 1:** Clinical and laboratory characteristics in 3694 subjects with a fatty liver index (FLI) <60 and 1598 subjects with an FLI ≥ 60

	FLI < 60 (N = 3694)	FLI ≥ 60 (N = 1598)	*P* value
Sex, n (%)			
Male	1523 (41.2%)	1048 (65.6%)	<.001
Age, mean (SD), y	51.7 (11.9)	56.9 (11.2)	<.001
BMI, mean (SD), kg/m^2^	24.8 (2.8)	30.9 (4.2)	<.001
Waist circumference, mean (SD), cm	85.9 (9.2)	105.3 (9.4)	<.001
SBP, mean (SD), mm Hg	121.6 (17.4)	135.1 (17.9)	<.001
DBP, mean (SD), mm Hg	71.4 (8.6)	77.3 (8.7)	<.001
History of Cancer, n (%)	179 (4.8%)	67 (4.2%)	.55
History of CVD, n (%)	106 (2.9%)	97 (6.1%)	<.001
Smoking status, n (%)			
Never	1147 (31.1%)	379 (23.7%)	<.001
Former	1440 (39.0%)	784 (49.1%)	
Current <6 cigarettes per day	175 (4.7%)	61 (3.8%)	
Current 6‐20 cigarettes per day	760 (20.6%)	275 (17.2%)	
Current >20 cigarettes per day	124 (3.4%)	78 (4.9%)	
Alcohol consumption, n (%)			
No, almost never	879 (23.8%)	461 (28.8%)	<.001
1‐4 drinks per month	653 (17.7%)	250 (15.6%)	
2‐7 drinks per week	1233 (33.4%)	442 (27.7%)	
1‐3 drinks per day	810 (21.9%)	347 (21.7%)	
>3 drinks per day	119 (3.2%)	98 (6.1%)	
Glucose lowering medication, n (%)	68 (1.8%)	120 (7.5%)	<.001
Antihypertensive medication, n (%)	525 (14.2%)	573 (35.9%)	<.001
Lipid lowering medication, n (%)	219 (5.9%)	221 (13.8%)	<.001
TMAO, μmol/L	2.99 (1.60, 5.46)	3.58 (2.02, 6.25)	<.001
Glucose, mmol/L	4.70 (4.40, 5.10)	5.20 (4.70, 5.90)	<.001
HOMA‐IR, median (IQR), mU mmol/L^2^/22.5	1.44 (1.05, 2.03)	3.06 (2.13, 4.67)	<.001
TC, mean (SD), mmol/L	5.28 (0.99)	5.72 (1.08)	<.001
HDL‐C, mean (SD), mmol/L	1.32 (0.31)	1.09 (0.24)	<.001
Triglycerides, median (IQR), mmol/L	0.95 (0.72, 1.27)	1.74 (1.31, 2.38)	<.001
ALT, median (IQR), U/L	15.0 (12.0, 20.0)	23.0 (17.0, 32.0)	<.001
AST, median (IQR), U/L	21.0 (19.0, 25.0)	25.0 (21.0, 30.0)	<.001
GGT, median (IQR), U/L	19.0 (14.0, 28.0)	41.0 (29.0, 63.0)	<.001
FLI, median (IQR), AU	22.56 (10.18, 38.99)	80.33 (70.03, 90.21)	<.001
HSI, median (IQR), AU	31.82 (29.34, 34.58)	39.25 (35.86, 43.02)	<.001
Plasma albumin, g/L	44.0 (42.0, 45.0)	44.0 (42.0, 45.0)	.10
eGFR, mean (SD), ml/min/1.73 m^2^	94.50 (16.39)	87.79 (17.62)	<.001
UAE, median (IQR), mg/24 h	7.81 (5.79, 12.82)	11.42 (7.08, 26.65)	<.001

Abbreviations: AU, arbitrary units; ALT, alanine aminotransferase; AST, aspartate aminotransferase; BMI, body mass index; DBP, diastolic blood pressure; eGFR, estimated glomerular filtration rate; FLI, fatty liver index; GGT, gamma‐glutamyltransferase; HDL‐C, high‐density lipoprotein cholesterol; HOMA, Homeostasis Model Assessment; HSI, hepatic steatosis index; SBP, systolic blood pressure; TC, total cholesterol; TMAO, trimethylamine‐N‐Oxide; UAE, urinary albumin excretion.

### 3.2 Cross‐sectional analyses

The association of the plasma concentrations of TMAO with baseline characteristics was evaluated with both univariable and multivariable linear regression analyses. In the univariable analyses, sex, systolic blood pressure, diastolic blood pressure, history of CVD, former smoking, antihypertensive medication, GGT, and plasma albumin were significantly associated with plasma concentrations of TMAO (Table 2). In a multivariable model, TMAO remained independently associated with age, BMI, waist circumference, alcohol consumption, medication for T2D, glucose, HOMA‐IR, HDL‐cholesterol, triglycerides, ALT, FLI, HSI, eGFR, and UAE (Table 2). To further evaluate the strength of the association of TMAO with NAFLD, FLI and HSI were evaluated in two separate multivariable models without the risk of multicollinearity (VIF < 5) that included the variables above mentioned. In such models, circulating concentrations of TMAO remained significantly associated with an elevated FLI (Std. β = 0.10 [95% CI 0.03, 0.17], *P =* .003) and an elevated HSI (Std. β = 0.14 [95% CI 0.08, 0.20], *P* < .001) (Tables S2 and S3).

**TABLE 2:** Univariable and multivariable associations of baseline characteristics with plasma concentrations of TMAO in 5292 PREVEND participants

	Univariable regression analysis		Multivariable regression analysis	
	Std. β (95% CI)	*P* value	Std. β (95% CI)	*P* value
Men, n	0.06 (0.00, 0.11)	.04	0.04 (−0.02, 0.09)	.20
Age, y	0.12 (0.10, 0.15)	<.001	0.12 (0.09, 0.14)	**<.001**
BMI, kg/m^2^	0.09 (0.07, 0.12)	<.001	0.07 (0.04, 0.10)	**<.001**
Waist circumference, cm	0.10 (0.08, 0.13)	<.001	0.08 (0.05, 0.11)	**<.001**
SBP, mm Hg	0.06 (0.03, 0.08)	<.001	0.00 (−0.03, 0.03)	.91
DBP, mm Hg	0.04 (0.01, 0.07)	.004	0.00 (−0.03, 0.03)	.88
History of cancer, n	−0.01 (−0.14, 0.12)	.89	−0.03 (−0.16, 0.10)	.65
History of CVD, n	0.19 (0.05, 0.33)	.008	0.07 (−0.07, 0.22)	.32
Smoking status, n				
Former	0.08 (0.02, 0.15)	.01	0.04 (−0.03, 0.11)	.23
Current <6 cig/d	0.06 (−0.08, 0.20)	.4	0.06 (−0.07, 0.20)	.37
Current 6‐20 cig/d	−0.05 (−0.13, 0.03)	.19	−0.06 (−0.13, 0.02)	.16
Current >20 cig/d	0.04 (−0.11, 0.18)	.61	0.05 (−0.09, 0.20)	.49
Alcohol consumption, n				
1‐4 drinks per month	0.01 (−0.07, 0.10)	.78	0.03 (−0.05, 0.12)	.47
2‐7 drinks per week	0.06 (−0.01, 0.13)	.10	0.10 (0.03, 0.17)	.**01**
1‐3 drinks per day	0.05 (−0.03, 0.13)	.19	0.06 (−0.02, 0.14)	.13
>3 drinks per day	0.17 (0.03, 0.31)	.02	0.17 (0.03, 0.32)	.**02**
Glucose lowering medication, n	0.37 (0.23, 0.52)	<.001	0.27 (0.13, 0.42)	**<.001**
Antihypertensive medication, n	0.12 (0.05, 0.19)	<.001	0.01 (−0.06, 0.08)	.76
Lipid lowering medication, n	0.14 (0.04, 0.24)	.005	0.05 (−0.05, 0.15)	.29
Glucose, mmol/L	0.08 (0.05, 0.11)	<.001	0.06 (0.03, 0.08)	**<.001**
HOMA‐IR, mU mmol/L^2^/22.5	0.08 (0.05, 0.11)	<.001	0.06 (0.03, 0.09)	**<.001**
TC, mmol/L	0.01 (−0.01, 0.04)	.31	−0.01 (−0.04, 0.02)	.56
HDL‐C, mmol/L	−0.05 (−0.07, −0.02)	<.001	−0.04 (−0.07, −0.01)	.**004**
Triglycerides, mmol/L	0.05 (0.02, 0.07)	.001	0.03 (0.00, 0.06)	.**02**
ALT, U/L	0.04 (0.01, 0.06)	.008	0.03 (0.01, 0.06)	.**01**
AST, U/L	0.02 (−0.01, 0.04)	.26	0.00 (−0.03, 0.03)	.90
GGT, U/L	0.04 (0.02, 0.07)	.002	0.03 (−0.00, 0.05)	.06
FLI, ≥60 AU	0.19 (0.13, 0.25)	<.001	0.14 (0.08, 0.21)	**<.001**
HSI, ≥36 AU	0.19 (0.13, 0.25)	<.001	0.16 (0.11, 0.22)	**<.001**
Plasma albumin, g/L	−0.04 (−0.06, −0.01)	.009	−0.02 (−0.05, 0.01)	.13
eGFR, ml/min/1.73 m^2^	−0.16 (−0.19, −0.13)	<.001	−0.15 (−0.19, −0.12)	**<.001**
UAE, mg/24 h	0.07 (0.04, 0.09)	<.001	0.06 (0.03, 0.08)	**<.001**

Note

Standardized beta regression coefficients (95% confidence intervals) are shown. Multivariable regression coefficients are adjusted for age and sex.

Abbreviations: AU, arbitrary units; ALT, alanine aminotransferase; AST, aspartate aminotransferase; BMI, body mass index; DBP, diastolic blood pressure; eGFR, estimated glomerular filtration rate; FLI, fatty liver index; GGT, gamma‐glutamyl transferase; HDL‐C, high‐density lipoprotein cholesterol; HOMA, homeostasis model assessment; HSI, hepatic steatosis index; SBP, systolic blood pressure; TC, total cholesterol; TMAO, trimethylamine‐N‐Oxide; UAE, urinary albumin excretion; β, standardized beta regression coefficient.

Although the circulating TMAO concentrations were higher in participants with NAFLD, in comparison with participants without NAFLD; the concentrations of TMAO remained negatively associated with eGFR in participants with and without NAFLD: (Std. β = −0.17 [95% CI −0.22, −0.11], *P* < .001) and (Std. β = −0.14 [95% CI −0.18, −0.09], *P* < .001), respectively (Figure S1).

### 3.3 Longitudinal analyses

In the all‐cause mortality analysis conducted in the whole population, there was a significant interaction between TMAO and NAFLD (*P*_int_ < .05); therefore, we conducted the analyses separately in the groups with and without NAFLD.

### 3.4 All‐cause mortality in subjects with NAFLD

**FIGURE 1:**
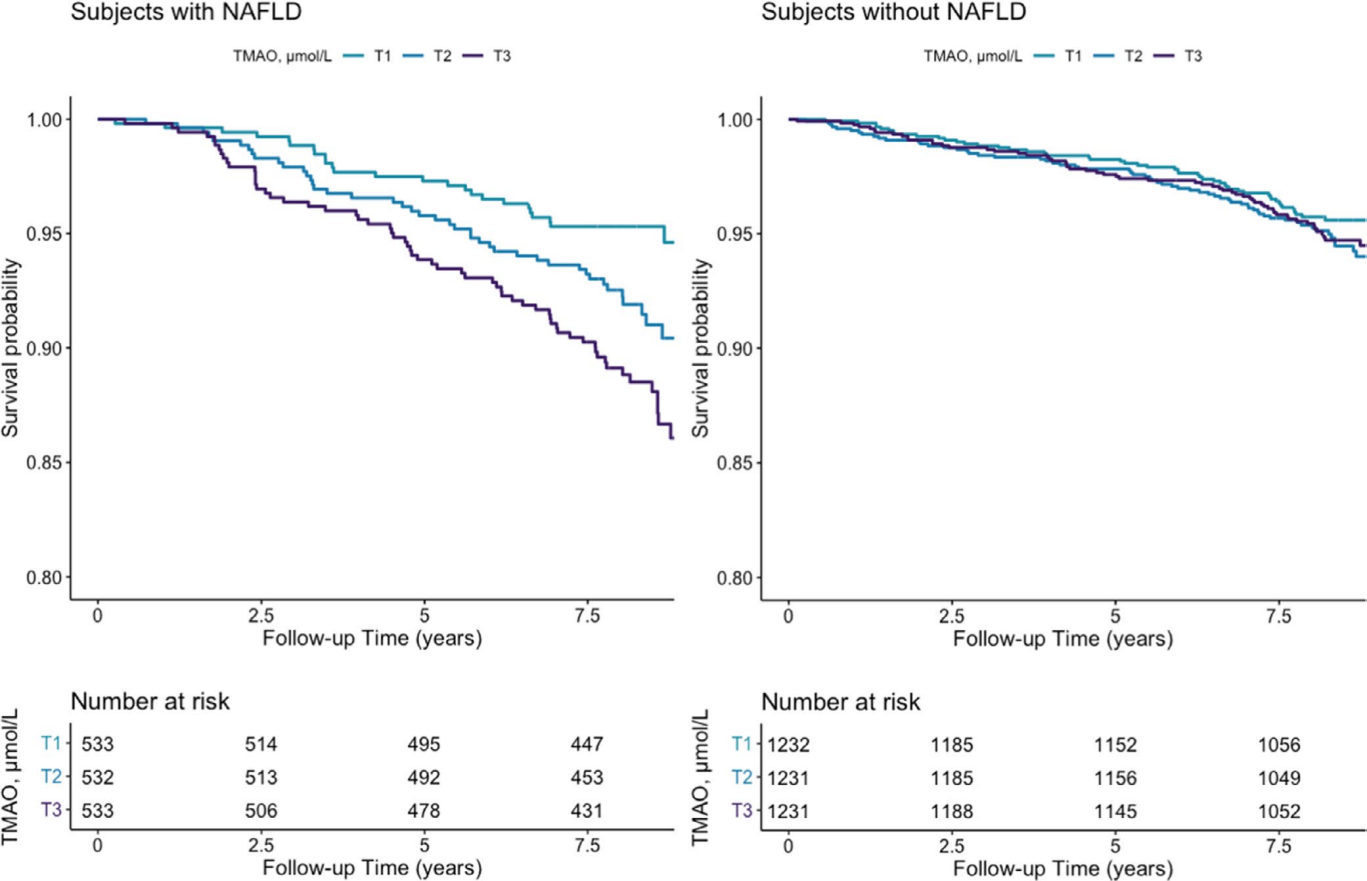
Kaplan–Meier plots for all‐cause mortality comparing tertiles of TMAO

After a median (IQR) follow‐up of 8.2 (5.7‐11.8) years, 133 deaths were recorded. Kaplan–Meier curves for all‐cause mortality according to tertiles of TMAO plasma concentration are presented in Figure 1. There was an increased risk of all‐cause mortality associated with the top tertile of TMAO concentrations (*P* for log‐rank test <.001).

**FIGURE 2:**
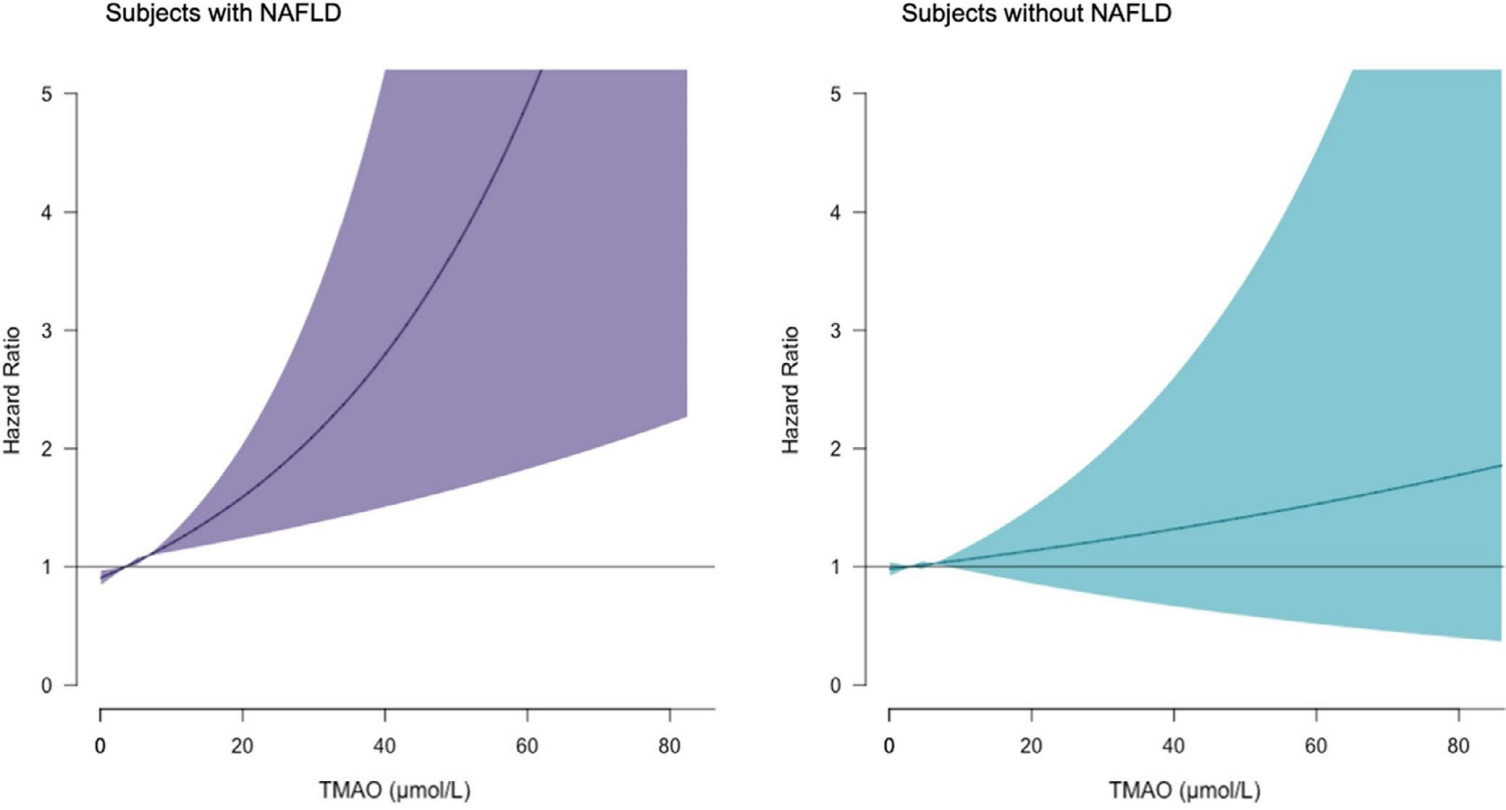
Association between circulating concentrations of TMAO and all‐cause mortality

In Cox proportional hazard regression analyses that examined the TMAO concentrations as HR per 1 Ln SD (Figure 2), increased plasma concentrations of TMAO were associated with increased risk of all‐cause mortality independent of age and sex (_adj_HR, 1.25 [95% CI 1.04, 1.49], *P* = .01; Model 1, Table 3); systolic blood pressure, smoking status, alcohol consumption, history of cancer, glucose lowering medication, and lipid lowering medication (_adj_HR, 1.24 [95% CI 1.04, 1.48], *P* = .02; Model 2, Table 3); total cholesterol, HDL‐cholesterol, and glucose (_adj_HR, 1.23 [95% CI 1.03, 1.47], *P* = .03; Model 3, Table 3); albuminuria and eGFR < 90 ml/min/1.73 m^2^ (_adj_HR, 1.21 [95% CI 1.01, 1.46], *P* = .04; Model 4, Table 3). The proportional hazards assumptions were not violated for any of the variables in the additive models. Analyses of plasma concentration of TMAO as a categorical variable, using the first tertile as the reference group, showed that the third tertile of TMAO plasma concentration was also associated with higher risk of all‐cause mortality in all the cumulative models described, resulting in a fully _adj_HR 1.90 (95% CI 1.18, 3.04), *P* = .008 (Model 4, Table 3). In the longitudinal analysis, there was a significant interaction of TMAO with eGFR (*P*_int_ < .01), but there was no significant interaction with age (*P*_int_ > .10). Similar results were obtained in the analysis using HSI as proxy of NAFLD (Table S4). According to the sensitivity analyses, to invalidate the inference about the association of TMAO with all‐cause mortality, 48.6% of the estimated effect would have to be due to bias. Likewise, in order to invalidate the inference, the effect of TMAO on all‐cause mortality should be equal to 0 in 777 out of the 1598 participants, which highlights the robustness of the association.

**TABLE 3:** Association of TMAO with all‐cause mortality, assessed with Cox proportional hazard ratios in subjects with NAFLD (FLI ≥ 60)

	TMAO per 1 Ln SD increment		T1	T2		T3	
Participants, n	1598		533	532		533	
Events, n	133		25	45		63	
	HR (95% CI)	*P* value		HR (95% CI)	*P* value	HR (95% CI)	*P* value
Crude model	1.41 (1.18, 1.69)	<.001	(ref)	1.80 (1.10, 2.93)	.01	2.55 (1.60, 4.05)	<.001
Model 1	1.25 (1.04, 1.49)	.01	(ref)	1.66 (1.02, 2.70)	.04	2.16 (1.36, 3.44)	.01
Model 2	1.24 (1.04, 1.48)	.02	(ref)	1.66 (1.02, 2.71)	.04	2.01 (1.26, 3.21)	.003
Model 3	1.23 (1.03, 1.47)	.03	(ref)	1.71 (1.04, 2.79)	.03	1.96 (1.23, 3.12)	.005
Model 4	1.21 (1.01, 1.46)	.04	(ref)	1.69 (1.03, 2.77)	.04	1.90 (1.18, 3.04)	.008

Note

Data are presented as hazard ratios (HRs) with 95% confidence intervals (CIs) and *P* values. Model 1: Model adjusted for age + sex. Model 2: Model 1 + SBP + smoking status + alcohol consumption + cancer history + glucose lowering medication + lipid lowering medication. Model 3: Model 2 + TC + HDL‐C + Glucose. Model 4: Model 3 + albuminuria + reduced eGFR (<90 ml/min/1.73 m^2^).

The association of TMAO with all‐cause mortality in NAFLD was also evaluated on sensitivity analysis after excluding subjects with high alcohol consumption (more than three drinks per day). Analyses of plasma concentration of TMAO as a categorical variable, using the first tertile as the reference group, showed that the third tertile of TMAO plasma concentration was associated with increased risk of all‐cause mortality (HR, 2.48 [95% CI 1.55, 3.99], *P* < .001) (Table S5); after adjustment for the above‐described variables, the TMAO plasma concentration remained associated with higher risk of all‐cause mortality, resulting in an adjusted _adj_HR 1.75 (95% CI 1.08, 2.84), *P* = .02 (Table S5).

Additionally, the association of TMAO with all‐cause mortality was also evaluated on sex‐specific groups. The association did not reach formal significance in women, having a crude HR 2.14 (95% CI 0.81, 5.64), *P* = .12. Men presented a crude HR 3.56 (95% CI 2.04, 6.21), *P* < .001 (Table S6). After adjustment for the above‐described variables, the associations of TMAO plasma concentration with risk of cardiovascular mortality remained consistent in both groups, having a fully _adj_HR 2.46 (95% CI 0.91, 6.13), *P* = .08 in women, and an _adj_HR of 2.17 (95% CI 1.23, 3.84), *P* = .007 in men (Table S6).

### 3.5 All‐cause mortality in subjects without NAFLD

In subjects without NAFLD, plasma concentrations of TMAO were not associated with an increased risk of all‐cause mortality (*P* for log‐rank test >.05) (Figure 1). In Cox proportional hazard regression analyses that examined the circulating TMAO concentrations as HR per 1 Ln SD, increased plasma concentrations of TMAO were not associated with increased risk of all‐cause mortality, neither in the crude model (HR, 1.14 [95% CI 0.98, 1.33], *P* = .09; Table 4) nor in the full model adjusted for age, sex, systolic blood pressure, smoking status, alcohol consumption, history of cancer, antidiabetic medication and lipid lowering medication, total cholesterol, HDL‐cholesterol, glucose, albuminuria, and reduced eGFR (_adj_HR, 1.07 [95% CI 0.91, 1.26], *P* = .42; Model 4, Table 4). Similarly, the analyses of plasma concentration of TMAO as a categorical variable, using the first tertile as the reference group, showed that the third tertile of TMAO plasma concentration was not associated with higher risk of all‐cause mortality in all the cumulative model described above, resulting in a fully _adj_HR 0.95 (95% CI 0.65, 1.39), *P* = .78 (Model 4, Table 4). Similar results were obtained in the analysis using HSI as proxy of NAFLD (Table S7).

**TABLE 4:** Association of TMAO with all‐cause mortality, assessed with Cox proportional hazard ratios in subjects without NAFLD (FLI < 60)

	TMAO per 1 Ln SD increment		T1	T2		T3	
Participants, n	3694		1232	1231		1231	
Events, n	174		51	62		61	
	HR (95% CI)	*P* value		HR (95% CI)	*P* value	HR (95% CI)	*P* value
Crude Model	1.14 (0.98, 1.33)	.09	(ref)	1.22 (0.84, 1.76)	.30	1.18 (0.81, 1.71)	.39
Model 1	1.02 (0.87, 1.20)	.81	(ref)	0.86 (0.59, 1.24)	.41	0.90 (0.62, 1.31)	.59
Model 2	1.07 (0.91, 1.26)	.41	(ref)	0.90 (0.62, 1.30)	.56	0.94 (0.65, 1.37)	.75
Model 3	1.07 (0.91, 1.25)	.42	(ref)	0.90 (0.62, 1.31)	.56	0.94 (0.65, 1.37)	.75
Model 4	1.07 (0.91, 1.26)	.42	(ref)	0.91 (0.62, 1.32)	.61	0.95 (0.65, 1.39)	.78

Note

Data are presented as hazard ratios (HRs) with 95% confidence intervals (CIs) and *P* values. Model 1: Model adjusted for age + sex. Model 2: Model 1 + SBP + smoking status + alcohol consumption + cancer history + glucose lowering medication + lipid lowering medication. Model 3: Model 2 + TC + HDL‐C + glucose. Model 4: Model 3 + albuminuria + reduced eGFR (<90 ml/min/1.73 m^2^).

### 3.6 Cardiovascular mortality in subjects with NAFLD

In subjects with NAFLD, the Cox proportional hazard regression analyses that examined the circulating TMAO concentrations as HR per 1 Ln SD, increased plasma concentrations of TMAO were associated with increased risk cardiovascular mortality, only in the crude model (HR, 1.49 [95% CI 1.04, 2.12], *P* = .02; Table S8) but not in the full adjusted model (_adj_HR, 1.14 [95% CI 0.80, 1.63], *P* = .48; Table S8). Similarly, the analyses of plasma concentration of TMAO as a categorical variable, using the first tertile as the reference group, showed that the third tertile of TMAO plasma concentration was associated with higher risk of cardiovascular mortality only in the crude model (HR, 4.20 [95% CI 1.58, 11.15], *P* = .003; Table S8) but not in the fully adjusted model (_adj_HR, 2.50 [95% CI 0.91, 6.81], *P* = .07; Table S8).

The association of TMAO with cardiovascular mortality in NAFLD was also evaluated on sensitivity analysis after excluding subjects with high alcohol consumption (more than three drinks per day). Analyses of plasma concentration of TMAO as a categorical variable, using the first tertile as the reference group, showed that the third tertile of TMAO plasma concentration was associated with increased risk of cardiovascular mortality (HR, 2.48 [95% CI 1.55, 3.99], *P* < .001) (Table S9); after adjustment for the above‐described variables, the TMAO plasma concentration remained associated with higher risk of cardiovascular mortality, resulting in a adjusted _adj_HR 1.75 (95% CI 1.08, 2.84), *P* = .02 (Table S9).

### 3.7 Cardiovascular mortality in subjects without NAFLD

In subjects without NAFLD, the Cox proportional hazard regression analyses that examined the circulating TMAO concentrations as HR per 1 Ln SD, increased plasma concentrations of TMAO were not associated with increased risk cardiovascular mortality, neither in the crude model (HR, 1.35 [95% CI 0.97, 1.86], *P* = .07; Table S10) nor in the full adjusted model (_adj_HR, 1.26 [95% CI 0.89, 1.79], *P* = .20; Table S10). Similarly, the analyses of plasma concentration of TMAO as a categorical variable, using the first tertile as the reference group, showed that the third tertile of TMAO plasma concentration was not associated with higher risk of cardiovascular mortality neither in the crude model (HR, 1.69 [95% CI 0.77. 3.69], *P* = .19; Table S10) nor in the fully adjusted model (_adj_HR, 1.30 [95% CI 0.58, 2.91], *P* = .52; Table S10).

## 4 DISCUSSION

In this prospective cohort, we have shown that higher plasma TMAO concentrations were significantly associated with an increased risk of all‐cause mortality in individuals with NAFLD, as judged by an FLI score ≥60. Importantly, such association was not present in subjects without NAFLD. Plasma concentrations of TMAO at baseline were higher in subjects with NAFLD, compared with those without NAFLD. Cross‐sectionally, TMAO was associated with several metabolic risk factors including adiposity, reduced eGFR, older age, and plasma glucose, as well as with the use of glucose lowering medication. The most relevant clinical and biochemical variables associated with TMAO reported in the present study and the literature are summarized in Table 5.^35^, ^36^ Notably, the association of TMAO with increased risk of all‐cause mortality was independent of these variables. These results are in line with previous studies that have shown that altered gut microbiota composition may control the rate of progression of multiple metabolic syndrome‐associated pathologies such as NAFLD.^6^

**TABLE 5:** Clinical and biochemical variables associated with TMAO

Clinical and biochemical variables associated with TMAO
Use of glucose lowering medication
Age
Fatty Liver Index
Estimated glomerular filtration rate
Alcohol consumption
Waist circumference
Body mass index
Vitamin D (reference 35)^a^
Glucose
Homeostasis model assessment of insulin resistance
High‐density lipoprotein cholesterol
Triglycerides
Reactive oxygen species (reference 36)^a^
Inteleukin 18 (reference 36)^a^

^a^ Denotes variables did not include in the present analysis.

In this study, NAFLD was assessed with two validated scores: FLI and HSI. Both scores were positively associated with plasma concentrations of TMAO. Our results are in accordance with a prior pilot study conducted in 137 subjects with metabolic syndrome (57% women), aged 21‐56 years. In that study, circulating TMAO, measured by means of high‐performance liquid chromatography–mass spectrometry, was linearly associated with FLI score values (β = 0.82, *P* < .001).^37^ Similarly, in a small case‐control study comprising 34 subjects with biopsy‐proven NAFLD, it was demonstrated that circulating TMAO concentrations were associated with a more advanced disease status.^15^ A more recent and larger study (n = 357, 76% women) also provided consistent evidence about the association of biopsy‐proven NAFLD with plasma concentrations of TMAO. In this report, higher concentrations of TMAO were also associated with worse clinical and histological characteristics in patients with NAFLD. Importantly, the authors found that circulating TMAO concentrations were not associated with hepatic *FMO3* expression, suggesting that the concentrations of TMAO are not mainly dependent on the human liver metabolism of TMAO, but rather on its clearance rate by the kidneys.^13^ Accordingly, we found that TMAO concentrations were strongly associated with eGFR at baseline (Figure S1) as previously reported.^12^ Likewise, in the prospective analysis of all‐cause mortality, we found that there was a significant interaction of TMAO with eGFR.

TMAO has been shown to play a role in the development of atherosclerosis, by promoting platelet hyperreactivity.^38^ Zhu et al demonstrated that circulating TMAO promotes a hyper response of platelets aggregation in response to thrombin, in an in vitro setting. They also demonstrated that TMAO promotes an increased platelet adhesion to collagen surfaces. Furthermore, in in vivo thrombosis assays, they have shown that the formation of thrombus after an arterial injury is enhanced in animals fed with TMAO‐enriched diets.^38^ Accordingly, we found that higher TMAO concentrations were associated cross‐sectionally with history of CVD. Nevertheless, the prospective analysis showed that elevated concentrations of TMAO were not particularly associated with an increased risk of cardiovascular mortality (Tables S6 and S7).

The mechanisms underlying the association between TMAO and all‐cause mortality in subjects with NAFLD remain to be investigated. Of note, previous studies conducted in subjects with NAFLD had shown that subject with high concentrations of circulating TMAO present a more advanced disease stage, characterized by more steatosis, hepatocellular ballooning, and lobular inflammation.^14^

The worsening of NAFLD associated to high TMAO might be caused by its effect on decreasing the bile acids pool.^14^ Some mechanism had been proposed: decreasing synthesis of bile acids due to the inhibition of the key enzymes CYP7A1 and CYP27A116^8^ and constraining the enterohepatic circulation of bile acids between the liver and the gut due to the repression of organic anion transporter family protein expression.^8^ Furthermore, there is evidence from experimental models of NAFLD on which it had been shown that TMAO increases the hepatic triglyceride accumulation by inhibition the farnesoid X receptor signalling.^15^

Future interventions to ameliorate the excess of mortality in patients with NAFLD with special focus in improving the microbiota deserve attention. Previous studies have reported that adherence to the Mediterranean diet, characterized by reduced consumption of animal‐derived protein, is associated with lower concentrations of TMAO.^39^ Furthermore, it has been reported that the micronutrient, vitamin D, was strongly associated to both NAFLD and TMAO concentrations;^35^ therefore, the study of nutritional interventions to explore the effect of micronutrient supplementation also merit further research.

NMR represents a methodology capable of offering high‐throughput metabolite quantifications in a cost‐effective manner, in comparison with other metabolomic technologies.^40^ Therefore, it is plausible that TMAO quantification by means of NMR could represent a useful tool for the evaluation of clinical interventions to ameliorate adverse cardiometabolic consequences of NAFLD.

### 4.1 Strengths and limitations

This study has strengths. First, this study comprises a long‐term follow‐up, and includes the record of several important confounders for the analysis of both all‐cause and cardiovascular mortality. In addition, the large population enrolled in the study enabled us to carry out sufficiently powered multivariable‐adjusted analyses and test the robustness of the findings using sensitivity analyses to provide solid evidence. In addition, the sample size also facilitates the generalization of our findings to similar populations; in fact, our cross‐sectional results were in agreement with a larger Dutch study that included a total of 37 496 participants.^41^ Furthermore, patients enrolled in the PREVEND cohort were followed‐up for a period of time that is long enough to allow us the study of metabolites with potentially subtle and cumulative effects, which seems to be the case of microbiota‐derived metabolites such as TMAO.^42^ Finally, to the best of our knowledge, this is the first study to report the association between elevated circulating TMAO concentrations and increased risk of all‐cause mortality in subjects with NAFLD. Several limitations of the present study deserve mentioning. First, the present study was conducted in the north of the Netherlands and mainly comprises individuals of Caucasian ancestry, which could limit the extrapolation of our findings to other ethnicities. In our report, categorization of NAFLD rely on the FLI and the HSI, which are proxies of NAFLD for which nuclear magnetic resonance imaging or liver biopsy are preferred diagnostic procedures in small scale studies. Similarly, alcohol consumption was self‐reported, and therefore, we cannot disregard the possibility of residual confounding due to imprecise reports. In addition, the observational nature of the study prevents us to draw causal conclusions. Nevertheless, the robust body of external experimental evidence would suggest a causal role of TMAO in the increased risk of mortality within NAFLD populations. Finally, it is worth mentioning that residual confounding is an important limitation of all observational studies.

In conclusion, we presented the first evidence about the increased risk of mortality associated to elevated concentrations of TMAO, in subjects with NAFLD. Such prospective association was independent of traditional risk factors and comorbidities. Further investigation is needed to determine if TMAO‐lowering interventions could improve the prognosis of patients with NAFLD.

## ETHICS APPROVAL AND CONSENT TO PARTICIPATE

The study conforms to the ethical guidelines of the 1975 Declaration of Helsinki and was approved by the local ethics committee of the University Medical Center Groningen (approval number: MEC96/01/022). All participants provided written informed consent.

## CONFLICT OF INTEREST

JLFG, PRD, AP, GN, RPFD, and SJLB declare that they have no competing interests. MAC and EG are employees of Labcorp.

## AUTHORS CONTRIBUTIONS

JLFG conceived the work, performed statistical analysis, and wrote the manuscript. PRD contributed to the drafting of the manuscript. AP contributed to the drafting of the manuscript. MAC performed the assay for TMAO and contributed to the drafting of the manuscript. EG performed the assay for TMAO and contributed to the drafting of the manuscript. GN contributed to drafting and revision of the manuscript. RPFD conceived the work, performed statistical analysis and wrote the manuscript. SJLB conceived the work, performed statistical analysis and wrote the manuscript. All authors critically revised the article and provided the final approval of the version to be published.

## Supporting information

Supplementary Material

## Data Availability

Data are available upon reasonable request due to privacy.
