# Supplementary material for: Circulating trimethylamine‐N‐oxide is associated with all‐cause mortality in subjects with nonalcoholic fatty liver disease
Source: Liver Int. 2021 Jun 8;41(10):2371–82. doi: 10.1111/liv.14963 (PMC8518486; doi:10.1111/liv.14963)
Supplement: Supplementary file 1 — Supplementary Material [file LIV-41-2371-s001.docx]

**SUPPLEMENTAL MATERIAL**

**Circulating TMAO is Associated with All-Cause Mortality in Subjects with Non-Alcoholic Fatty Liver Disease.**

Jose L. Flores-Guerrero^1*^, Adrian Post^1^, Peter R. van Dijk^2^, Margery A. Connelly^4^, Erwin Garcia^4^, Gerjan Navis^1^, Stephan J.L. Bakker^1^, Robin P.F. Dullaart^2^

^1^ Department of Internal Medicine, Division of Nephrology, University of Groningen, University Medical Center Groningen, Groningen, The Netherlands.

^2^ Department of Internal Medicine, Division of Endocrinology, University of Groningen, University Medical Center Groningen, Groningen, The Netherlands.

^3^ Laboratory Corporation of America Holdings (LabCorp), Morrisville, North Carolina, USA.

*Corresponding author: Jose L. Flores-Guerrero, email address: j.l.flores.guerrero[@umcg.nl](mailto:j.l.flores.guerrero@umcg.nl), ORCID: 0000-0002-6094-2206

**Supplemental Table 1.** STROBE Statement—Checklist of items that should be included in reports of cohort studies

|  | Item No | Recommendation | Page No |
| --- | --- | --- | --- |
| Title and abstract | 1 | (a) Indicate the study’s design with a commonly used term in the title or the abstract | 2 |
|  |  | (b) Provide in the abstract an informative and balanced summary of what was done and what was found | 2 |
| Introduction | | | |
| Background/rationale | 2 | Explain the scientific background and rationale for the investigation being reported | 4 |
| Objectives | 3 | State specific objectives, including any prespecified hypotheses | 4 |
| Methods | | | |
| Study design | 4 | Present key elements of study design early in the paper | 5 |
| Setting | 5 | Describe the setting, locations, and relevant dates, including periods of recruitment, exposure, follow-up, and data collection | 5 |
| Participants | 6 | (a) Give the eligibility criteria, and the sources and methods of selection of participants. Describe methods of follow-up | 5 |
|  |  | (b) For matched studies, give matching criteria and number of exposed and unexposed |  |
| Variables | 7 | Clearly define all outcomes, exposures, predictors, potential confounders, and effect modifiers. Give diagnostic criteria, if applicable | 7,8 |
| Data sources/ measurement | 8* | For each variable of interest, give sources of data and details of methods of assessment (measurement). Describe comparability of assessment methods if there is more than one group | 6,7 |
| Bias | 9 | Describe any efforts to address potential sources of bias | 7,8 |
| Study size | 10 | Explain how the study size was arrived at | 5 |
| Quantitative variables | 11 | Explain how quantitative variables were handled in the analyses. If applicable, describe which groupings were chosen and why | 5, 7 |
| Statistical methods | 12 | (a) Describe all statistical methods, including those used to control for confounding | a.7-8 |
|  |  | (b) Describe any methods used to examine subgroups and interactions | b.NA |
|  |  | (c) Explain how missing data were addressed | c.5 |
|  |  | (d) If applicable, explain how loss to follow-up was addressed | d.NA |
|  |  | (e) Describe any sensitivity analyses | e.8, 10 |
| Results | | |  |
| Participants | 13* | (a) Report numbers of individuals at each stage of study—eg numbers potentially eligible, examined for eligibility, confirmed eligible, included in the study, completing follow-up, and analysed | 5 |
|  |  | (b) Give reasons for non-participation at each stage | 5 |
|  |  | (c) Consider use of a flow diagram |  |
| Descriptive data | 14* | (a) Give characteristics of study participants (eg demographic, clinical, social) and information on exposures and potential confounders | 20 |
|  |  | (b) Indicate number of participants with missing data for each variable of interest | 5 |
|  |  | (c) Summarise follow-up time (eg, average and total amount) | 10 |
| Outcome data | 15* | Report numbers of outcome events or summary measures over time | 22 |
| Main results | 16 | (a) Give unadjusted estimates and, if applicable, confounder-adjusted estimates and their precision (eg, 95% confidence interval). Make clear which confounders were adjusted for and why they were included | 22 |
|  |  | (b) Report category boundaries when continuous variables were categorized | 22, 22 |
|  |  | (c) If relevant, consider translating estimates of relative risk into absolute risk for a meaningful time period | 10, 11 |
| Other analyses | 17 | Report other analyses done—eg analyses of subgroups and interactions, and sensitivity analyses | 10 |
| Discussion |  |  |  |
| Key results | 18 | Summarise key results with reference to study objectives | 12 |
| Limitations | 19 | Discuss limitations of the study, taking into account sources of potential bias or imprecision. Discuss both direction and magnitude of any potential bias | 14 |
| Interpretation | 20 | Give a cautious overall interpretation of results considering objectives, limitations, multiplicity of analyses, results from similar studies, and other relevant evidence | 12-14 |
| Generalisability | 21 | Discuss the generalisability (external validity) of the study results | 13-14 |
| Other information |  |  |  |
| Funding | 22 | Give the source of funding and the role of the funders for the present study and, if applicable, for the original study on which the present article is based | 15 |

*Give information separately for exposed and unexposed groups.

**Supplemental Fig 1.** Association of Trimethylamine N-oxide with eGFR in participants with and without NAFLD.


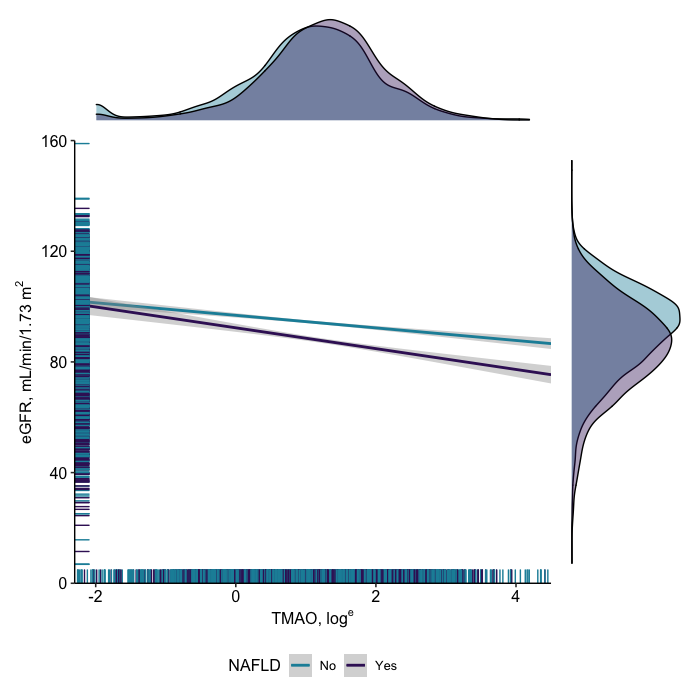


**Supplemental Table 2.** Multivariable associations of baseline characteristics, excluding HSI and its determinants, with plasma concentrations of TMAO in 5292 PREVEND participants

| **Characteristic** | **Std β** | **95% CI** | ***p* value** |
| --- | --- | --- | --- |
| Men, n | 0.02 | -0.04, 0.08 | 0.46 |
| Age, years | 0.01 | -0.03, 0.05 | 0.64 |
| SBP, mmHg | -0.04 | -0.08, 0.01 | 0.11 |
| DBP, mmHg | 0.01 | -0.03, 0.05 | 0.53 |
| History of CVD, n | 0.02 | -0.13, 0.17 | 0.78 |
| Glucose lowering medication, n | 0.15 | -0.01, 0.31 | 0.07 |
| Antihypertensive medication, n | -0.04 | -0.11, 0.04 | 0.33 |
| Lipid lowering medication, n | -0.03 | -0.13, 0.08 | 0.60 |
| Glucose, mmol/L | 0.04 | 0.01, 0.07 | **0.004** |
| HDL-C, mmol/L | -0.01 | -0.04, 0.02 | 0.66 |
| FLI, ≥ 60 A. U. | 0.10 | 0.03, 0.17 | **0.003** |
| Plasma albumin, g/L | -0.02 | -0.05, 0.01 | 0.19 |
| eGFR, mL/min/1.73 m^2^ | -0.15 | -0.18, -0.11 | **<0.001** |
| UAE, mg/24 h | 0.03 | 0.01, 0.06 | **0.02** |

Standardized beta regression coefficients (95% Confidence Intervals) are shown.

Abbreviations. β, standardized beta regression coefficient, A.U., arbitrary units, DBP, diastolic blood pressure, eGFR, estimated glomerular filtration rate, FLI, fatty liver index, HDL-C, high-density lipoprotein cholesterol, HSI, hepatic steatosis index, SBP, systolic blood pressure, TMAO, Trimethylamine N-Oxide, UAE, urinary albumin excretion.

**Supplemental Table 3.** Multivariable associations of baseline characteristics, excluding FLI and its determinants, with plasma concentrations of TMAO in 5292 PREVEND participants

| **Characteristic** | **Std β** | **95% CI** | ***p* value** |
| --- | --- | --- | --- |
| Men, n | 0.05 | -0.01, 0.11 | 0.12 |
| Age, years | 0.01 | -0.03, 0.05 | 0.57 |
| SBP, mmHg | -0.04 | -0.08, 0.00 | 0.07 |
| DBP, mmHg | 0.01 | -0.03, 0.05 | 0.48 |
| History of CVD, n | 0.03 | -0.12, 0.17 | 0.74 |
| Glucose lowering medication, n | 0.14 | -0.02, 0.30 | 0.10 |
| Antihypertensive medication, n | -0.04 | -0.11, 0.04 | 0.32 |
| Lipid lowering medication, n | -0.03 | -0.13, 0.08 | 0.59 |
| Glucose, mmol/L | 0.04 | 0.01, 0.07 | **0.010** |
| HDL-C, mmol/L | 0.00 | -0.04, 0.03 | 0.79 |
| HSI, ≥ 36 A. U. | 0.14 | 0.08, 0.20 | **<0.001** |
| Plasma albumin, g/L | -0.02 | -0.04, 0.01 | 0.21 |
| eGFR, mL/min/1.73 m^2^ | -0.15 | -0.19, -0.11 | **<0.001** |
| UAE, mg/24 h | 0.04 | 0.01, 0.06 | **0.01** |

Standardized beta regression coefficients (95% Confidence Intervals) are shown.

Abbreviations. β, standardized beta regression coefficient, A.U., arbitrary units, DBP, diastolic blood pressure, eGFR, estimated glomerular filtration rate, FLI, fatty liver index, HDL-C, high-density lipoprotein cholesterol, HSI, hepatic steatosis index, SBP, systolic blood pressure, TMAO, Trimethylamine N-Oxide, UAE, urinary albumin excretion.

**Supplemental Table 4.** Association of TMAO with all-cause mortality, assessed with Cox Proportional Hazard ratios in subjects with NAFLD (HSI ≥ 36).

|  | **TMAO per 1 Ln  SD Increment** | | **T1** | **T2** | | **T3** | |
| --- | --- | --- | --- | --- | --- | --- | --- |
| Participants, *n* | 1756 | | 586 | 585 | | 585 | |
| Events, *n* | 109 | | 21 | 30 | | 58 | |
|  | HR (95 % CI) | *p* value |  | HR (95 % CI) | *p* value | HR (95 % CI) | *p* value |
| Crude Model | 1.52 (1.24,1.86) | <0.001 | (ref) | 1.41 (0.81,2.47) | 0.22 | 2.81 (1.71,4.63) |  |
| Model 1 | 1.36 (1.11,1.67) | 0.003 | (ref) | 1.16 (0.66,2.02) | 0.60 | 2.20 (1.33,3.63) | 0.002 |
| Model 2 | 1.35 (1.10,1.65) | 0.004 | (ref) | 1.19 (0.68,2.09) | 0.54 | 2.17 (1.31,3.58) | 0.003 |
| Model 3 | 1.32 (1.07,1.63) | 0.008 | (ref) | 1.20 (0.68,2.10) | 0.53 | 2.03 (1.23,3.36) | 0.006 |
| Model 4 | 1.27 (1.03,1.57) | 0.03 | (ref) | 1.14 (0.65,1.99) | 0.66 | 1.83 (1.10,3.07) | 0.02 |

Data are presented as hazard ratios (HRs) with 95% confidence intervals (CIs) and *p* values.

Model 1. Model adjusted for age +sex

Model 2. Model 1 + SBP + Smoking status + alcohol consumption + cancer history + glucose lowering medication + lipid lowering medication

Model 3. Model 2 + TC + HDL-C + Glucose

Model 4. Model 3 + albuminuria + reduced eGFR (< 90 mL/min/1.73 m^2^)

**Supplemental** **Table 5.** Association of TMAO with all-cause mortality, assessed with Cox Proportional Hazard ratios in subjects with NAFLD (FLI > 60), after exclusion of subjects with high alcohol consumption (>3 drinks per day).

|  | **TMAO per 1 Ln  SD Increment** | | **T1** | **T2** | | **T3** | |
| --- | --- | --- | --- | --- | --- | --- | --- |
| Participants, *n* | 1497 | | 499 | 499 | | 499 | |
| Events, *n* | 127 | | 24 | 44 | | 59 | |
|  | HR (95 % CI) | *p* value |  | HR (95 % CI) | *p* value | HR (95 % CI) | *p* value |
| Crude Model | 1.39 (1.16,1.68) | <0.001 | (ref) | 1.84 (1.12,3.03) | 0.01 | 2.48 (1.55,3.99) | <0.001 |
| Model 1 | 1.23 (1.02,1.47) | 0.03 | (ref) | 1.54 (0.94,2.53) | 0.09 | 1.98 (1.23,3.19) | 0.004 |
| Model 2 | 1.22 (1.01,1.48) | 0.03 | (ref) | 1.60 (0.97,2.63) | 0.06 | 1.86 (1.15,3.00) | 0.01 |
| Model 3 | 1.22 (1.01,1.47) | 0.04 | (ref) | 1.63 (0.99,2.69) | 0.05 | 1.79 (1.11,2.90) | 0.02 |
| Model 4 | 1.21 (1.00,1.45) | 0.05 | (ref) | 1.61 (0.98,2.66) | 0.06 | 1.75 (1.08,2.84) | 0.02 |

Data are presented as hazard ratios (HRs) with 95% confidence intervals (CIs) and *p* values.

Model 1. Model adjusted for age

Model 2. Model 1 + SBP + Smoking status + alcohol consumption + cancer history + glucose lowering medication + lipid lowering medication

Model 3. Model 2 + TC + HDL-C + Glucose

Model 4. Model 3 + albuminuria + reduced eGFR (< 90 mL/min/1.73 m^2^)

**Supplemental** **Table 6.** Association of TMAO with all-cause mortality, assessed with Cox Proportional Hazard ratios in men and women with NAFLD (FLI > 60) separately.

| **Women** | **TMAO per 1 Ln  SD Increment** | | **T1** | **T2** | | **T3** | |
| --- | --- | --- | --- | --- | --- | --- | --- |
| Participants, *n* | 550 | | 184 | 183 | | 183 | |
| Events, *n* | 27 | | 6 | 8 | | 13 | |
|  | HR (95 % CI) | *p* value |  | HR (95 % CI) | *p* value | HR (95 % CI) | *p* value |
| Crude Model | 1.34 (0.90,2.01) | 0.15 | (ref) | 1.35 (0.47,3.89) | 0.57 | 2.14 (0.81,5.64) | 0.12 |
| Model 1 | 1.36 (0.89,2.07) | 0.15 | (ref) | 1.22 (0.42,3.52) | 0.71 | 2.25 (0.85,5.91) | 0.10 |
| Model 2 | 1.39 (0.91,2.13) | 0.13 | (ref) | 1.24 (0.43,3.58) | 0.69 | 2.30 (0.86,6.14) | 0.10 |
| Model 3 | 1.40 (0.91,2.16) | 0.12 | (ref) | 1.19 (0.41,3.50) | 0.74 | 2.37 (0.88,6.37) | 0.09 |
| Model 4 | 1.43 (0.93,2.20) | 0.10 | (ref) | 1.25 (0.42,3.71) | 0.69 | 2.46 (0.91,6.63) | 0.08 |
| **Men** | **TMAO per 1 Ln  SD Increment** | | **T1** | **T2** | | **T3** | |
| Participants, *n* | 1048 | | 350 | 350 | | 349 | |
| Events, *n* | 106 | | 16 | 35 | | 55 | |
|  | HR (95 % CI) | *p* value |  | HR (95 % CI) | *p* value | HR (95 % CI) | *p* value |
| Crude Model | 1.46 (1.19,1.79) | <0.001 | (ref) | 2.17 (1.20,3.92) | 0.01 | 3.56 (2.04,6.21) | <0.001 |
| Model 1 | 1.23 (1.01,1.50) | 0.04 | (ref) | 1.83 (1.01,3.30) | 0.05 | 2.55 (1.46,4.45) | 0.001 |
| Model 2 | 1.22 (0.99,1.49) | 0.05 | (ref) | 1.91 (1.06,3.47) | 0.03 | 2.40 (1.37,4.21) | 0.002 |
| Model 3 | 1.18 (0.97,1.45) | 0.09 | (ref) | 1.95 (1.07,3.55) | 0.03 | 2.26 (1.29,3.98) | 0.004 |
| Model 4 | 1.17 (0.95,1.44) | 0.13 | (ref) | 1.93 (1.06,3.52) | 0.03 | 2.17 (1.23,3.84) | 0.007 |

Data are presented as hazard ratios (HRs) with 95% confidence intervals (CIs) and *p* values.

Model 1. Model adjusted for age

Model 2. Model 1 + SBP + Smoking status + alcohol consumption + cancer history + glucose lowering medication + lipid lowering medication

Model 3. Model 2 + TC + HDL-C + Glucose

Model 4. Model 3 + albuminuria + reduced eGFR (< 90 mL/min/1.73 m^2^)

**Supplemental Table 7.** Association of TMAO with all-cause mortality, assessed with Cox Proportional Hazard ratios in subjects without NAFLD (FLI < 60).

|  | **TMAO per 1 Ln  SD Increment** | | **T1** | **T2** | | **T3** | |
| --- | --- | --- | --- | --- | --- | --- | --- |
| Participants, *n* | 3536 | | 1179 | 1178 | | 1179 | |
| Events, *n* | 198 | | 54 | 71 | | 73 | |
|  | HR (95 % CI) | *p* value |  | HR (95 % CI) | *p* value | HR (95 % CI) | *p* value |
| Crude Model | 1.16 (0.99,1.34) | 0.05 | (ref) | 1.32 (0.93,1.88) | 0.12 | 1.33 (0.93,1.88) | 0.12 |
| Model 1 | 1.02 (0.87,1.18) | 0.83 | (ref) | 0.98 (0.68,1.39) | 0.89 | 1.00 (0.70,1.43) | 0.99 |
| Model 2 | 1.04 (0.90,1.21) | 0.58 | (ref) | 0.98 (0.69,1.41) | 0.93 | 0.98 (0.68,1.39) | 0.89 |
| Model 3 | 1.04 (0.90,1.21) | 0.58 | (ref) | 1.00 (0.70,1.42) | 0.98 | 0.98 (0.69,1.40) | 0.90 |
| Model 4 | 1.05 (0.91,1.23) | 0.50 | (ref) | 1.01 (0.71,1.45) | 0.95 | 1.01 (0.71,1.45) | 0.96 |

Data are presented as hazard ratios (HRs) with 95% confidence intervals (CIs) and *p* values.

Model 1. Model adjusted for age +sex

Model 2. Model 1 + SBP + Smoking status + alcohol consumption + cancer history + glucose lowering medication + lipid lowering medication

Model 3. Model 2 + TC + HDL-C + Glucose

Model 4. Model 3 + albuminuria + reduced eGFR (< 90 mL/min/1.73 m^2^)

**Supplemental** **Table 8.** Association of TMAO with cardiovascular mortality, assessed with Cox Proportional Hazard ratios in subjects with NAFLD (FLI ≥ 60).

|  | **TMAO per 1 Ln  SD Increment** | | **T1** | **T2** | | **T3** | |
| --- | --- | --- | --- | --- | --- | --- | --- |
| Participants, *n* | 1598 | | 533 | 532 | | 533 | |
| Events, *n* | 36 | | 5 | 10 | | 21 | |
|  | HR (95 % CI) | *p* value |  | HR (95 % CI) | *p* value | HR (95 % CI) | *p* value |
| Crude Model | 1.49 (1.04,2.12) | 0.02 | (ref) | 1.99 (0.68,5.82) | 0.20 | 4.20 (1.58,11.15) | 0.003 |
| Model 1 | 1.26 (0.90,1.77) | 0.17 | (ref) | 1.78 (0.61,5.21) | 0.29 | 3.32 (1.25,8.81) | 0.01 |
| Model 2 | 1.20 (0.85,1.69) | 0.29 | (ref) | 1.96 (0.67,5.74) | 0.22 | 2.90 (1.08,7.79) | 0.03 |
| Model 3 | 1.16 (0.81,1.65) | 0.43 | (ref) | 1.94 (0.66,5.75) | 0.23 | 2.56 (0.94,6.93) | 0.07 |
| Model 4 | 1.14 (0.80,1.63) | 0.48 | (ref) | 1.97 (0.66,5.86) | 0.22 | 2.50 (0.91,6.81) | 0.07 |

Data are presented as hazard ratios (HRs) with 95% confidence intervals (CIs) and *p* values.

Model 1. Model adjusted for age +sex

Model 2. Model 1 + SBP + Smoking status + alcohol consumption + cancer history + glucose lowering medication + lipid lowering medication

Model 3. Model 2 + TC + HDL-C + Glucose

Model 4. Model 3 + albuminuria + reduced eGFR (< 90 mL/min/1.73 m^2^)

**Supplemental** **Table 9.** Association of TMAO with cardiovascular mortality, assessed with Cox Proportional Hazard ratios in subjects with NAFLD (FLI > 60), after exclusion of subjects with high alcohol consumption (>3 drinks per day).

|  | **TMAO per 1 Ln  SD Increment** | | **T1** | **T2** | | **T3** | |
| --- | --- | --- | --- | --- | --- | --- | --- |
| Participants, *n* | 1497 | | 499 | 499 | | 499 | |
| Events, *n* | 35 | | 5 | 10 | | 20 | |
|  | HR (95 % CI) | *p* value | (ref) | HR (95 % CI) | *p* value | HR (95 % CI) | *p* value |
| Crude Model | 1.46 (1.02,2.09) | 0.04 | (ref) | 2.01 (0.69,5.87) | 0.20 | 3.99 (1.50,10.64) | 0.005 |
| Model 1 | 1.22 (0.87,1.73) | 0.25 | (ref) | 1.66 (0.57,4.85) | 0.35 | 2.94 (1.10,7.85) | 0.03 |
| Model 2 | 1.16 (0.81,1.65) | 0.42 | (ref) | 1.80 (0.61,5.28) | 0.29 | 2.58 (0.95,6.98) | 0.06 |
| Model 3 | 1.12 (0.77,1.61) | 0.56 | (ref) | 1.84 (0.62,5.44) | 0.27 | 2.28 (0.83,6.23) | 0.11 |
| Model 4 | 1.11 (0.77,1.60) | 0.58 | (ref) | 1.87 (0.63,5.58) | 0.26 | 2.24 (0.81,6.17) | 0.12 |

Data are presented as hazard ratios (HRs) with 95% confidence intervals (CIs) and *p* values.

Model 1. Model adjusted for age

Model 2. Model 1 + SBP + Smoking status + alcohol consumption + cancer history + glucose lowering medication + lipid lowering medication

Model 3. Model 2 + TC + HDL-C + Glucose

Model 4. Model 3 + albuminuria + reduced eGFR (< 90 mL/min/1.73 m^2^)

**Supplemental** **Table 10.** Association of TMAO with cardiovascular mortality, assessed with Cox Proportional Hazard ratios in subjects without NAFLD (FLI < 60).

|  | **TMAO per 1 Ln  SD Increment** | | **T1** | **T2** | | **T3** | |
| --- | --- | --- | --- | --- | --- | --- | --- |
| Participants, *n* | 3694 | | 1232 | 1231 | | 1231 | |
| Events, *n* | 43 | | 10 | 16 | | 17 | |
|  | HR (95 % CI) | *p* value |  | HR (95 % CI) | *p* value | HR (95 % CI) | *p* value |
| Crude Model | 1.35 (0.97,1.86) | 0.07 | (ref) | 1.60 (0.73,3.53) | 0.24 | 1.69 (0.77,3.69) | 0.19 |
| Model 1 | 1.17 (0.83,1.65) | 0.38 | (ref) | 0.98 (0.44,2.16) | 0.95 | 1.15 (0.52,2.52) | 0.72 |
| Model 2 | 1.26 (0.89,1.79) | 0.20 | (ref) | 1.08 (0.48,2.41) | 0.85 | 1.31 (0.59,2.91) | 0.50 |
| Model 3 | 1.27 (0.89,1.80) | 0.19 | (ref) | 1.05 (0.47,2.35) | 0.91 | 1.33 (0.60,2.94) | 0.49 |
| Model 4 | 1.26 (0.89,1.79) | 0.20 | (ref) | 1.03 (0.46,2.32) | 0.93 | 1.30 (0.58,2.91) | 0.52 |

Data are presented as hazard ratios (HRs) with 95% confidence intervals (CIs) and *p* values.

Model 1. Model adjusted for age +sex

Model 2. Model 1 + SBP + Smoking status + alcohol consumption + cancer history + glucose lowering medication + lipid lowering medication

Model 3. Model 2 + TC + HDL-C + Glucose

Model 4. Model 3 + albuminuria + reduced eGFR (< 90 mL/min/1.73 m^2^)
